# A Retrospective Study (2019–2023) on the Prevalence and Antimicrobial Resistance of Isolates from Canine Clinical Samples Submitted to the University Veterinary Hospital in Stara Zagora, Bulgaria

**DOI:** 10.3390/microorganisms12081670

**Published:** 2024-08-14

**Authors:** Velina Dinkova, Nikolina Rusenova

**Affiliations:** Department of Veterinary Microbiology, Infectious and Parasitic Diseases, Faculty of Veterinary Medicine, Trakia University, 6000 Stara Zagora, Bulgaria; velina.dinkova@yahoo.com

**Keywords:** canine isolates, skin, otitis, prevalence, antimicrobial resistance, multidrug resistance

## Abstract

The identification of local susceptibility patterns is important for the elaboration of effective local antimicrobial use guidelines and improvement in treatment outcomes. This retrospective study investigated the prevalence of microbial pathogens in dogs over a five-year period (2019–2023) and their antimicrobial resistance patterns with an emphasis on multidrug-resistant strains on the basis of 896 swab samples submitted to the microbiological laboratory at the University Veterinary Hospital, Stara Zagora, Bulgaria. A total of 1247 strains—1046 bacteria and 201 yeasts—were isolated. An increased proportion of *Staphylococcus* spp. as an agent of infections in dogs along with significant decrease in the share of *Streptococcus* spp. (from 16.2% in 2019 to 7.7% in 2023) was found. The occurrence of *Staphylococcus* spp. in otitis externa increased from 53.4% in 2019 to 84.5% in 2023 (*p* < 0.0001). The resistance of *Staphylococcus* spp. isolates to amoxicillin/clavulanic acid and cephalexin increased significantly in 2023 vs. 2022. At the same time, increased susceptibility to amikacin was observed in 2023 vs. 2019. For *Enterobacteriaceae*, significantly decreased resistance against amikacin and marbofloxacin was demonstrated in 2023 compared to 2019. Multidrug resistance (MDR) was present in 405 of 1046 bacterial isolates (38.7%). More than 50% of streptococci and pseudomonads were MDR. Of the MDR staphylococci, 41.7% were isolated from skin lesions and 28.3% were isolated from otitis. More than half of the strains resistant to seven, eight and nine groups of antimicrobial drugs (AMDs) were from wounds/abscesses. The results highlighted the importance of regular local monitoring of the spread of bacterial strains in veterinary clinics and their susceptibility to AMDs with regard to successful therapy outcomes and control on MDR spread.

## 1. Introduction

Dogs are the most popular companion animal species with definite positive impact on physical and psychological human health, in particular on the proper social development of children [1]. On the other hand, dogs are recognised as a reservoir for antibiotic-resistant microorganisms, as confirmed by reported high rates of multidrug resistance [2]. A meta-analysis of the occurrence of the ESKAPE bacteria group (*Enterococcus faecium, Staphylococcus aureus, Klebsiella pneumoniae*, *Acinetobacter baumannii*, *Pseudomonas aeruginosa* and *Enterobacter* spp.) in dogs involved in animal-assisted therapies confirmed a risk of bidirectional zoonotic transmission from dogs to humans [3]. This becomes increasingly important because while resistant bacteria appear quickly after treatment with antibiotics, their elimination even in the absence of selective pressure is slow [4].

Dogs play an important role in *Staphylococcus* spp. epidemiology, being carriers mainly at the skin and mucous membrane levels [5]. In this animal species, most bacterial infections are caused by the normal resident microflora of the skin and mucous membranes, mainly opportunistic *Staphylococcus* spp. and *S. pseudintermedius* in particular [6]. *Escherichia coli* (*E. coli*) and *Pseudomonas aeruginosa* (*P. aeruginosa*) are also recently reported as agents of secondary skin infections in dogs [7,8].

Antibiotics are essential for the treatment of infectious bacterial diseases in companion animals. In a clinical setting, antimicrobial therapy is in most cases empirical rather than based on antimicrobial susceptibility tests. The correct approach, however, is to prescribe antimicrobial drugs (AMDs) on the basis of data from bacteriological analysis, especially in chronic or recurrent infections.

The reported significant association between antimicrobial resistance (AMR) with the specific geographic region suggesting the role of local factors justifies its investigation. Thus, a study of temporal trends and predictors of antimicrobial resistance among canine *Staphylococcus* spp. isolates from swabs submitted to a diagnostic laboratory in the USA [9] affirmed that AMR was more common in urban areas with a higher number of dogs. The identification of local susceptibility patterns is important for the development of effective local antimicrobial use guidelines and improvement in treatment outcomes along with control on the development of antimicrobial resistance [7,10,11].

Previous studies conducted in Bulgaria reported data on the species composition and antimicrobial resistance of Gram-positive and Gram-negative bacteria limited to dogs with otitis externa [12,13,14]. More recent information on pathogenic microflora incriminated in the aetiology of canine skin and mucous coats infections in our country is not available, which was the incentive of performing the present study.

This retrospective study was conducted to analyse archival data (2019–2023) from the microbiological laboratory of the University Veterinary Hospital in Stara Zagora, Bulgaria, to investigate the spread of microbial pathogens associated with infections in dogs and to determine their antimicrobial drug resistance with emphasis on multidrug-resistant strains.

## 2. Materials and Methods

### 2.1. Ethics Statement

This study was conducted with the kind permission of the manager of the University Veterinary Hospital. All personal data were handled confidentially.

### 2.2. Samples

This study was performed on 896 swab samples from dogs with clinically manifested inflammation, submitted for analysis to the microbiological laboratory of the University Veterinary Hospital, Faculty of Veterinary Medicine, Trakia University in Stara Zagora, Bulgaria, from January 2019 to December 2023. The samples of ear, nasal, conjunctival, throat discharges, wound, abscess and fistula discharges, and fluid from skin lesions were obtained with sterile cotton-tipped applicators, immediately transferred to Stuart transport medium (Deltalab, Spain) and processed within 24 h.

### 2.3. Microbiological Examination and Identification of Isolates

Collected samples were plated on blood agar with 5% defibrinated sheep blood, MacConkey agar as selective and differential plating medium for *Enterobacteriaceae* and Sabouraud agar with 4% dextrose and 0.05 g/L chloramphenicol for yeasts (HiMedia, Mumbai, India). The samples were aerobically incubated for 18–24 h at 37 °C. After the initial incubation, the plates were checked for the presence of pathogenic bacteria. If no visible bacterial or yeast growth was detected, incubation continued for another 24 h at 37 °C. Microorganism presence was reported based on isolation in pure culture or as the predominant isolate in a mixed culture.

The identification of bacterial pathogens was based on clinical veterinary microbiology guidelines [15] on the basis of conventional microbiological tests specific to the respective suspected isolates. Briefly, the diagnostic algorithm for Gram-positive cocci included Gram staining, catalase and oxidase activity tests, and additional tests if necessary. Gram-negative isolates were subcultured from polytropic Kligler iron agar (KIA, HiMedia, India) and on medium indicating hydrogen sulphide production, motility and indole (SIM medium, HiMedia, India). The yeasts were identified according to the specific growth morphology of colonies and cell morphology [15].

### 2.4. Antimicrobial Susceptibility Testing

Antimicrobial susceptibility testing, which included 11 classes of antimicrobial drugs (AMDs), was performed using the disk diffusion susceptibility test [16]. The choice of drugs in each specific case depended on the isolates and clinical use. Penicillin was represented by an amoxicillin/clavulanic acid combination (20 + 10 µg). First-generation cephalosporins were represented by cephalexin (30 µg), third-generation cephalosporins were represented by cefovecin (30 µg) (Mastdiscs AST, Bootle, UK), and fourth-generation cephalosporins were represented by cefquinome (30 µg) (Biolab, Budapest, Hungary). The tested fluoroquinolones included enrofloxacin (5 µg), marbofloxacin (5 µg) (Biolab, Hungary), levofloxacin (5 µg); aminoglycosides: gentamicin (10 µg), amikacin (30 µg), neomycin (30 µg) and tobramycin (10 µg); macrolides: clarithromycin (15 µg) and azithromycin (15 µg), lincosamides: lincomycin (2 µg) and clindamycin (2 µg); tetracycline: doxycycline (30 µg), amphenicol: chloramphenicol (30 µg); polypeptide: colistin (10 µg); ansamycin: rifampin (5 µg); and sulphonamide: sulfamethoxazole/trimethoprim (23.75/1.25 µg). The discs were produced by HiMedia (India) unless otherwise indicated.

The reference strains *Staphylococcus aureus* ATCC 25923, *E. coli* ATCC 25922 and *P. aeruginosa* ATCC 27853 were used for internal quality control of antimicrobial disk susceptibility test zones according to the CLSI guidelines [16].

The strains were categorised as sensitive or resistant to the drugs in line with the approved guidelines [16]. Those with intermediate susceptibility were regarded as resistant. Isolates that showed non-susceptibility to at least one agent in three or more antimicrobial drug classes were accepted as multidrug-resistant (MDR) [17].

### 2.5. Data Analysis

The information from the database was organised in electronic tables to perform descriptive analyses by year of isolation, sampling site (skin, ears, nose, eyes, throat and wound discharge), bacterial groups (*Staphylococcus* spp., *Streptococcus* spp., *Pseudomonas* spp., *Enterobacteriaceae* and others) and antimicrobial drug susceptibility (sensitive or resistant). The level of statistical significance among the different bacterial groups and samples was evaluated with the chi-square test and tests for independent proportions (MedCalc v.15.8). The Cochran-Armitage test for trends was used to assess the year-wise alterations in antimicrobial resistance rates. *p*-values < 0.05 were considered statistically significant.

## 3. Results

### 3.1. Microorganisms Associated with Infections in Dogs

A total of 896 swab samples were analysed during the studied 5-year period. Of them, 18.4% were negative for microbial growth. From the other 731 swabs, 1247 strains were isolated: 1046 bacterial and 201 fungal identifications (from the *Malassezia* spp.). Year-wise distribution of samples over the 5 years showed that the number of samples submitted for analysis increased gradually over the years and in 2023 was almost 3 times higher than in 2019 (291 vs. 108).

The results demonstrated that 66% of all isolates belonged to *Staphylococcus* spp. (n = 690). The *Streptococcus* spp. was 6 times less frequently detected (n = 113). Gram-negative pathogens were from the *Enterobacteriaceae* family (n = 117) and non-fermentative bacteria from the *Pseudomonas* spp. (n = 69) (Table 1). Year-wise distribution of groups of microbial pathogens showed that the number of staphylococci increased 3 times from 2019 to 2023, but their proportion from all bacterial isolates for each year varied between 58.8% (2019) and 69% (2023). At the same time, the share of streptococci decreased significantly: from 16.2% in 2019 to 7.7% in 2023 (*p* = 0.0108).

According to sampling site (Table 2), almost half of strains were from otitis externa (n = 601), followed by isolates from skin lesions (n = 366) and wounds/abscesses (n = 135). About two-thirds of Gram-negative pathogens (*E. coli*, *Proteus* spp. and *Pseudomonas* spp.) were isolated from ear and skin samples. The majority of *Streptococcus* spp. was isolated from ears, skin, wounds and eyes (Table 2). Of all yeasts, 189 were from dogs with otitis externa and only 12 from skin lesions. Often, mixed infections with bacteria and *Malassezia* spp. yeasts were detected: from 52% of samples in 2019 to 71% in 2023.

**Figure 1:**
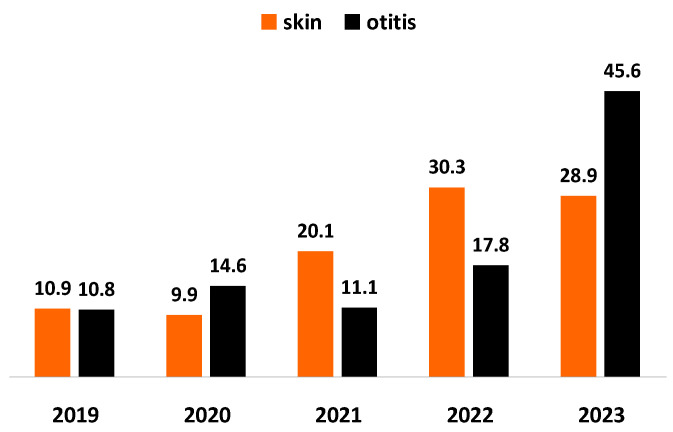
Year-wise prevalence of *Staphylococcus* spp. from all bacteria isolated from dogs with skin lesions and otitis externa for the 5-year study period.

*Staphylococcus* spp. was outlined as the predominating bacterial agent of otitis and skin inflammations in tested specimens. For the study period, the proportion of staphylococci from all pathogens involved in skin inflammations increased almost 3 times: from 10.9% in 2019 to 28.9% in 2023 and their share from infectious agents of otitis: almost 4 times (*p* < 0.0001), from 10.8% in 2019 to 45.6% in 2023 (Figure 1).

### 3.2. Patterns of Antimicrobial Drug Resistance

The resistance of bacterial isolates to most commonly tested antimicrobial drugs (AMDs) is presented in Table 3.

Statistically significant temporal changes in resistance rates over the 5-year study period in *Staphylococcus* spp. isolates were found in respect to colistin (*p* = 0.0002); amikacin (*p* = 0.0145); gentamicin (*p* = 0.0209); doxycycline (*p* = 0.0335), cephalexin (*p* = 0.0276) and in *Enterobacteriaceae* strains: with cefquinome (*p* = 0.0111); amikacin (*p* = 0.0056); enrofloxacin (*p* = 0.0379). The other bacterial groups (streptococci and pseudomonads) did not show consistent trends in susceptibility to the tested AMDs for the 5-year study period (Tables S1 and S2). *Pseudomonas* spp. isolates exhibited a substantial trend towards change in resistance only with regard to cefquinome (*p* = 0.0082).

At the end of the study period, significant increase in resistance rates of *Staphylococcus* spp. isolates against colistin (*p* = 0.0013 between 2019 and 2023) and doxycycline (*p* = 0.0097 between 2019 and 2022) was found out, whereas the resistance against amikacin declined in 2023 in relation to 2019 (*p* = 0.0177). As for the susceptibility to the fourth-generation cephalosporin cefquinome, it remained relatively high (Table 4). The representatives of *Enterobacteriaceae* demonstrated reduced resistance against the antimicrobial drugs applied most commonly in infections caused by members of this family. In comparison to 2019, the resistance rates against amikacin (*p* = 0.0291) and marbofloxacin (*p* = 0.05) in 2023 were statistically significantly lower (Table 5).

**Figure 2:**
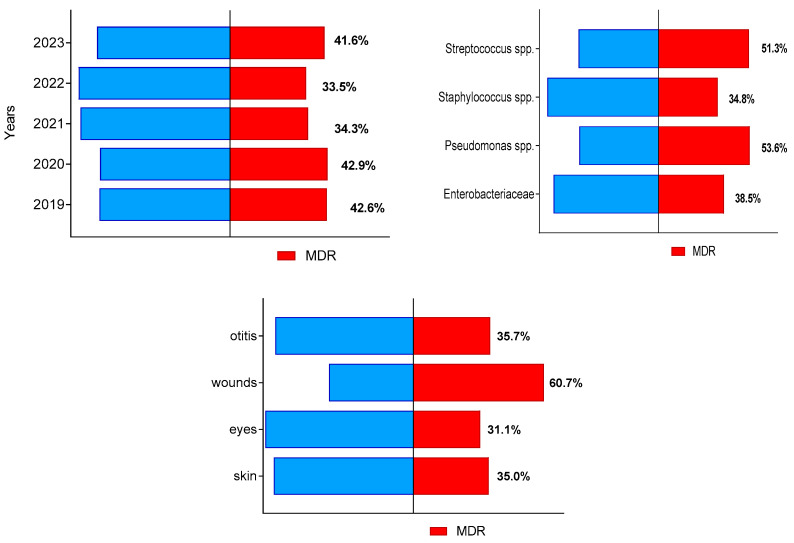
Distribution of canine multidrug-resistant (MDR) isolates by year of study, bacterial group and sample origin.

During the study period, a total of 405 of all 1046 bacterial isolates (38.7%; 95% CI 35.0–42.7%) were identified as multidrug-resistant (MDR). Their prevalence from all isolates in each of study years varied between 33.5% in 2022 and 42.9% in 2020 (Figure 2). The highest number of MDR isolates from all bacteria of the respective group were found out for *Pseudomonas* spp. (37 out of 69) and *Streptococcus* spp. (60 out of 113), followed by members of family *Enterobacteriaceae* and *Staphylococcus* spp. The highest proportion of MDR isolates (60.7%) were detected among isolates from suppurating wounds and abscesses (Figure 2).

**Figure 3:**
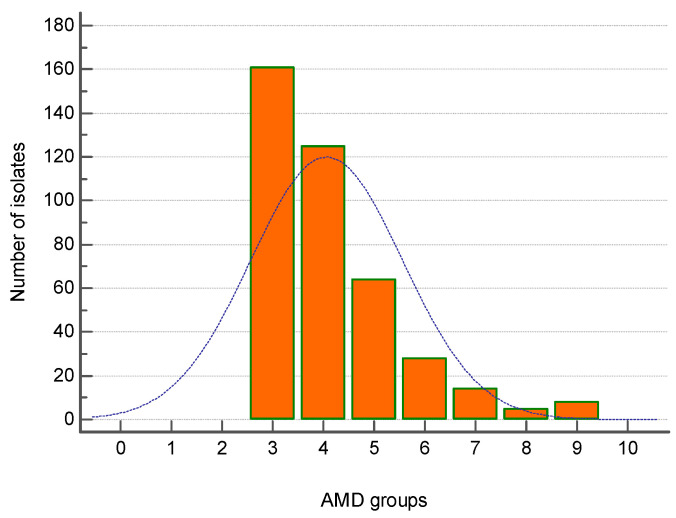
Distribution of all canine multidrug-resistant (MDR) isolates (n = 405) by number of groups of antimicrobial drugs (AMDs) to which they were resistant.

The majority of MDR strains (161 out of 405) were resistant to three groups of antimicrobial drugs (AMDs), followed by 124 strains resistant to four AMD groups and 64 isolates—to five AMD groups. Eight isolates were resistant to nine AMD groups (Figure 3).

**Figure 4:**
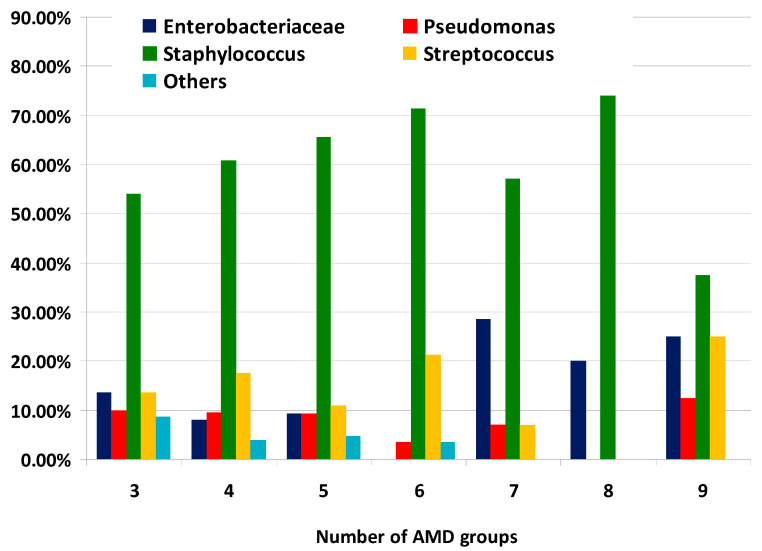
Distribution of types of canine multidrug-resistant (MDR) isolates by number of groups of antimicrobial drugs (AMDs) to which they were resistant.

The distribution of MDR bacterial groups according to the number of AMD groups to which they were resistant is illustrated on Figure 4. It should be noted that the proportions of *Enterobacteriaceae* representatives from all MDR strains resistant to seven, eight and nine AMD groups were greater than percentages demonstrating resistance to three, four and five AMD groups.

**Figure 5:**
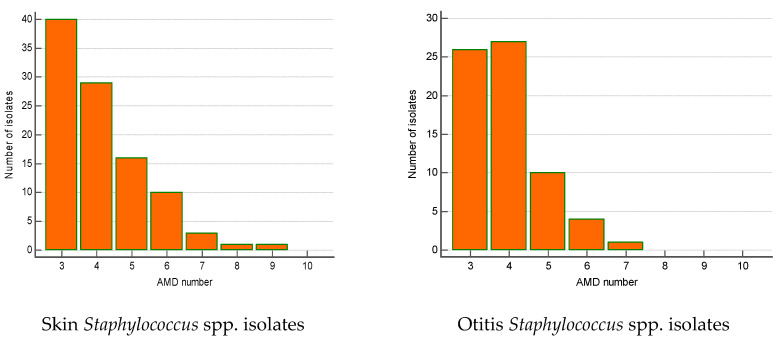
Number of MDR *Staphylococcus* spp. isolates from skin lesions and otitis by number of groups of antimicrobial drugs (AMDs) to which they were resistant.

The majority of MDR isolates belonged to *Staphylococcus* spp. (n = 240). Their number increased significantly by 23% over the 5-year period (from 12.1% in 2019 to 35% in 2023; *p* = 0.002). Most of MDR staphylococci were isolated from skin lesions (41.7%), ear discharge (28.3%) and wounds/abscesses (21.2%). The skin MDR staphylococci were most commonly resistant to three AMD groups (n = 40), four AMD groups (n = 29) and five AMD groups (n = 16). Some of them were resistant even to 8–9 AMD groups. A similar tendency was found out for MDR *Staphylococcus* spp. from otitis externa: predominance of strains resistant to three (n = 26), four (n = 27) and five AMD groups (n = 10) (Figure 5).

**Figure 6:**
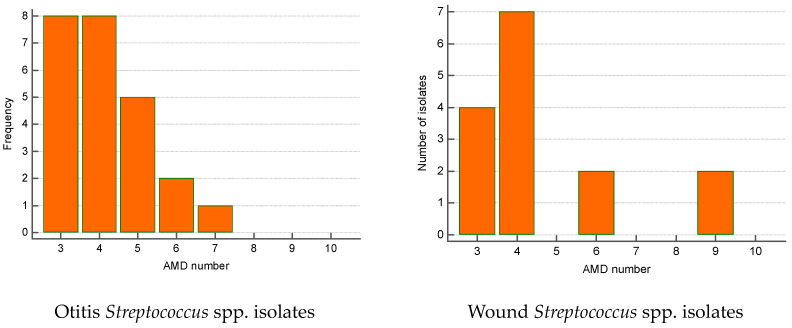
Number of MDR *Streptococcus* spp. isolates from skin lesions and otitis by number of groups of antimicrobial drugs (AMDs) to which they were resistant.

**Figure 7:**
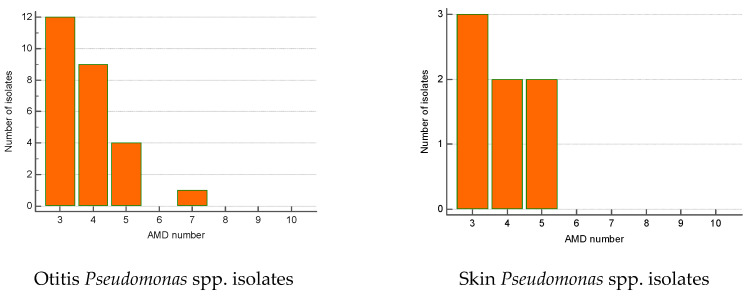
Number of MDR *Pseudomonas* spp. isolates from skin lesions and otitis by number of groups of antimicrobial drugs (AMDs) to which they were resistant.

The greatest proportion of MDR *Streptococcus* spp. isolates were recovered from otitis externa (40%), followed by wounds/abscesses (25%) and conjunctival discharge (16.7%). Seventy percent of *Pseudomonas* spp. isolates were from otitis, second came strains from skin lesions (18.9%). Regarding the number of AMD groups to which isolates showed resistance, otitis isolates of both genera were most commonly resistant to three and four groups of AMDs. The majority of wound *Streptococcus* spp. MDR strains were resistant to four groups of AMDs; two isolates were resistant to nine antimicrobials (Figure 6 and Figure 7).

The origin and multi-resistance patterns of isolates resistant to seven, eight and nine AMD groups (a total of 27 isolates) are presented in Table S3. More than half of these strains were from wounds/abscesses.

## 4. Discussion

In many veterinary clinics, antimicrobial drugs are mainly used empirically and the use of antimicrobial susceptibility testing (AST) is generally limited to chronic cases with poor response to first-line therapy [18]. However, the changes in AMR and the constant emergence of new agents and MDR phenotypes necessitate to obtain up-to-date information regarding the microorganisms involved in most commonly encountered canine pathologies and their antimicrobial susceptibility to achieve a positive outcome. In this respect, data from diagnostic veterinary laboratories may be extremely useful for outlining current trends in the prevalence and antimicrobial susceptibility of the microorganisms [9,19].

This study reports data on the prevalence of the commonest bacteria isolated from canine patients from 2019 to 2023 and their susceptibility to the main prescribed antibiotics. Year-wise distribution of samples over the 5 years showed that the number of submitted samples increased continuously and in 2023 was almost 3 times greater compared to 2019 (291 vs. 108). Similar tendencies were reported from different veterinary hospitals in Europe [6,7] and South America [10]. The notable increase in the number of samples submitted for bacteriological examinations in the last year of the present study compared with the previous years is encouraging as it confirms the increasing awareness of veterinary specialists about the benefits of bacteriological analysis before antimicrobial therapy prescription.

The majority of isolates in our study were bacterial (83.9%; 95% CI 78.9–89.1%). *Malassezia* spp. yeasts were also isolated (n = 201 or 16.1%; 95% CI 13.9–18.5), of them 189 were from dogs with otitis externa and 12 from skin lesions. *Malassezia* spp. is a commensal yeast that is normally present in low numbers in the external ear canals and superficial muco-cutaneous sites in dogs [20]. Co-infections of bacteria and *Malassezia* spp. were found out in 10.2% of 138 dogs [2]. A recent study conducted in Serbian veterinary clinics reported that *Malassezia pachydermatis* was the only isolated yeast pathogen in 68% of tested canine otitis swabs [21]. In Bulgaria, high prevalence of *M. pachydermatis* in dogs with otitis externa was also found out in previous years: 25% [12], 17% [13], 30% [14]. All these data confirm once again the necessity of detecting the presence of yeasts as pathogenic agents and inclusion of antifungal treatment in positive cases in order to avoid proliferation of yeasts after the antibacterial therapy and possibly, emergence of yeast dermatitis or otitis.

Most of Gram-positive and Gram-negative infections in dogs are caused by members of the normal resident microflora of the skin, mucous membrane and gastrointestinal tract. Of them, *Staphylococcus* spp. was the most frequently isolated bacterial genus confirming its role as opportunistic skin and mucous membrane pathogen. In this study, the proportion of *Staphylococcus* spp. from all bacterial pathogens varied from 58.8% in 2019 to 69% in 2023. *Staphylococcus* spp. turned out to be the main agent of otitis and skin inflammations. Its proportion from all bacterial agents of skin diseases demonstrated a three-fold increase from 10.9% in 2019 to 28.9% in 2023, whereas its percentage from all bacterial otitis pathogens increased more than 4 times (*p* < 0.0001): from 10.8% in 2019 to 45.6% in 2023. Staphylococci are repeatedly confirmed as the leading agents of otitis externa, pyoderma and postoperative wound infections in companion animals [22,23]. Ten years ago in Bulgaria, Petrov et al. [12] reported that coagulase-positive staphylococci were found in more than 70% (169/241) of dogs with bacterial otitis externa. Later data from Bulgaria demonstrated that coagulase-positive staphylococci were the dominating bacterial species in canine otitis externa: 31.5% (95% CI 19.9–44.4%) in 2010–2014 [13] and 36.83% (95% CI 33.1–40.8) for 2013–2017 [14]. Results about bacterial isolates from 1256 dogs and 94 cats examined in a veterinary diagnostic laboratory in Columbia over a 4-year period (2016–2019) showed similar findings: 58.6% of all strains belonged to *Staphylococcus* genus [10]. Our findings are consistent also with more recent reports, which define these bacteria as the most common isolates from dogs with otitis externa and pyoderma [24].

In this study, the percentage of *Pseudomonas* spp. from all bacterial pathogens tended to decrease slightly from 9.6% in 2019 to 5.8% in 2023. According to sampling site, this genus was found out in 10.4% of all otitis samples and 9.4% of conjunctival samples. *P. aeruginosa* was outlined as the second most common isolated bacteria from canine skin infections [6] and is reported among the four major pathogens in dogs with otitis [25]. Earlier studies in Bulgaria in dogs with otitis externa report higher prevalence rates of this pathogenic species than ours: 17% [12], 29.6% [13] and 16.24% [14]. Although *P. aeruginosa* is not a typical constituent of the canine ear microbiota, it is frequently isolated from cases of chronic otitis externa because the nature of this pathogen often makes treatment challenging [26].

In this study, the members of the family *Enterobacteriaceae* were responsible for 17.9% of skin infections, 15.3% of external otitis and 14.1% of wounds/abscesses. Compared to data provided by Petrov et al. [12] in dogs with otitis (17% *Proteus mirabilis*; 11% *E. coli*), a certain lower prevalence was detected in this study. Another investigation at a national scale has identified *P. mirabilis* as agent of 12.9% of canine otitis cases [13]. For the period 2013–2017, *Enterobacteriaceae* strains from Bulgarian dogs with otitis have decreased: 3.6% *P. mirabilis* and 3.2% *E. coli* [14]. Higher prevalence among canine otitis agents was reported from Pakistan: *E. coli* (13.55%), *Proteus* spp. (9.32%) [27].

The occurrence only of *Е. coli* in infected wounds from small animals in Slovakia was 13.88% [28], a rate similar to the prevalence reported in surgical site infections in small animals in Brazil [29].

Nowadays, humans and companion animals share most antibiotic-resistant bacteria and genes and thus play a crucial role in the emergence of antimicrobial resistance [4]. The most important findings of this study included increased resistance of staphylococci to colistin (*p* = 0.0013 between 2019 and 2023) and doxycycline (*p* = 0.0097 between 2019 and 2022) and increased susceptibility to amikacin (*p* = 0.0177 between 2019 and 2023). In relation to 2019, the resistance of *Enterobacteriaceae* isolates in 2023 to amikacin (*p* = 0.0291) and marbofloxacin (*p* = 0.05) has decreased. This finding may be interpreted as positive, giving that amikacin is a potential treatment option in infections caused by multidrug-resistant *S. pseudintermedius* (MRSP) [30].

In Bulgaria, over a 5-year period (2010–2014), Terziev and Urumova [13] reported that 81.3% of *P. aeruginosa* isolates exhibited sensitivity to gentamicin and 68.8% to enrofloxacin. Out of coagulase-positive staphylococci, 17.6% were resistant to ampicillin, amoxicillin/clavulanic acid and gentamicin. Resistance to enrofloxacin was found in 31.2% of pseudomonads and resistance to ampicillin and aminoglycosides was found in 14.2% of *P. mirabilis*. For 2013–2017, the antibacterial resistance patterns of isolates from Bulgarian dogs with otitis were characterised with increased resistance of coagulase-positive staphylococci and β-haemolytic streptococci to amoxicillin/clavulanic acid (42% and 50%, respectively) and gentamicin (29%, 40%). The lowest resistance rates of *P. aeruginosa* were detected against gentamicin (15%) and amikacin (18%). The percentage of pseudomonads resistant to fluoroquinolones was higher (27% against enrofloxacin and 35% against marbofloxacin) [14].

Our survey for 2019–2023 found out a low rate of resistance of *Pseudomonas* spp. against gentamicin (5.8%) in line with the retrospective study of Nocera et al. [6] in Italy that detected low levels of resistance of *P. aeruginosa* to gentamicin and marbofloxacin. Regarding enrofloxacin and marbofloxacin, our rates were 32.3% and 31.1%, respectively, similar to those reported by Ludwig et al. [31] and lower than rates between 40 and 100% to enrofloxacin of Nocera et al. [6]. In agreement with our results, the lowest levels of resistance in Italy were observed for gentamicin and marbofloxacin [6] along with high levels of resistance of *E. coli* and the other *Enterobacterales* strains to beta-lactams. A recent study from Poland [32] on the most common pathogens infecting wounds of companion animals and their antibiotic resistance demonstrated that *Enterobacterales* were mostly resistant to amoxicillin/clavulanic acid (68.3% of strains). This was not observed in our survey, where the overall resistance rate to amoxicillin/CA was 19.3% (95% CI 13.0–27.7%). It should be noted that *Enterobacteriaceae* isolates showed a low resistance to fluoroquinolones, mainly to levofloxacin and marbofloxacin, which is appropriate for treatment of otitis externa and pyoderma in pets. This is commented as a reassuring result [6], provided that fluoroquinolones are considered critically important antibiotics in human medicine.

The analysis of antimicrobial susceptibility data gathered between 2015 and 2021 from 38 German small animal practices [33] showed that resistance rates of *Staphylococcus* spp. (*S. pseudintermedius*, *S. aureus)* to amoxicillin/clavulanic acid and cefovecin ranged between 10% and 16%. Cefovecin is a third-generation cephalosporin antibiotic used to treat skin and soft tissue infections in dogs and cats. In our study, the resistance rate of *Staphylococcus* spp. to cefovecin was greater: 34.3% (95% CI 17.2–61.5%). In the study of Lenart-Boron et al. [32], 39.2% of staphylococci were resistant to clindamycin, whereas resistance rate to enrofloxacin in tested staphylococci was 11.8%. The respective rates of our study were again higher: 59.6% and 28.7%.

The susceptibility to antimicrobials of bacterial isolates from dogs (n = 1256) and cats (n = 94) in a veterinary diagnostic laboratory in Columbia (2016–2019) reported that all *Staphylococcus* spp. showed a moderate to high (20–50%) resistance to ampicillin, cephalosporin, enrofloxacin, gentamicin, tetracycline and trimethoprim-sulphonamide [10]. Comparable to our results, the members of this genus maintained a low (1–10%) resistance to amoxicillin/clavulanic acid. Overall, the antibiotics with the highest percentages of AMR for canine *Staphylococcus* spp. in the Valencia region [34] were from the penicillin group (almost 50%), chloramphenicol (≈50%), erythromycin (≈47%), clindamycin (≈40%) and tetracycline (≈40%). The AMR of quinolones was around 30%, varying from one *Staphylococcus* species to another, similarly to our results. Another antibiotic with high AMR found in the study from Spain was lincomycin, with the highest resistance in commensal *S. aureus* (83.3%) and *S. pseudintermedius* (25.9%).

A study conducted in dogs from the Iberian Peninsula [7] demonstrated that the tested *Streptococcus* spp. isolates presented the highest resistance percentage against amikacin and neomycin (>50%), which was comparable to our 5-year resistance rates (64% against amikacin and 60% against neomycin). The streptococcal isolates in this study were highly susceptible to amoxicillin/clavulanate (<10% resistance); a finding corresponding to the resistance rate of 13.5% of our survey and the rate of 3.3% reported by Bourély et al. [25]. Contrary to the preserved sensitivity to fluoroquinolones enrofloxacin and marbofloxacin (<20% resistance) making them effective for first-line therapy on the Iberian Peninsula [7], our resistance rates to tested fluoroquinolones were worrying: 81.8% against enrofloxacin, 85.9% against marbofloxacin and 94.4% against orbifloxacin. Data collected between 2012 and 2016 by the French national surveillance network for AMR [35] estimated the resistance level to fluoroquinolones in *Streptococcus* spp. from canine otitis at 62.9% (95% CI 59.8–65.9). The data from the present study confirm earlier data from our country regarding resistance to enrofloxacin in this species [14], giving indication that the use of fluoroquinolones for empirical treatment of streptococcal infections should be avoided.

The observed ascending or descending temporal trends in resistance to individual antimicrobial drugs reported from different countries may be attributed to usage frequency of a specific drug depending on varying clinical efficacy and the resulting higher or lower selection pressure.

The identification of MDR is important for treatment outcome and cost in veterinary patients as well as for public health due to the risk from transmission of resistance genes within pets, owners and veterinarians in a broader sense [19].

The main finding associated with MDR in the present study was the relatively high proportion of isolates from dogs resistant to three or more classes of AMDs: 405 of all 1046 bacterial strains for the 5-year period (38.7%). The highest proportion of MDR strains in relation to the total number of strains from the respective group was detected among *Pseudomonas* spp. (37 out of 69; 53.6%) and *Streptococcus* spp. (60 out of 113; 51.3%).

Nocera et al. [6] reported that among Gram-negative bacteria, *Pseudomonas aeruginosa* presented the highest values of antibiotic resistance with a higher share of MDR strains than ours: 79% (23/29). This may be attributed to the intrinsic resistance of *Pseudomonas* spp. to many antibiotics, the speed the bacteria become resistant and the remarkable collection of invasion and survival strategies [35]. Opposing to these data, MDR *P. aeruginosa* from canine otitis in the study of Rosales et al. [19] had a rate of 39/102 (38.2%), although the overall rate of MDR phenotypes was 47% (199/421; 95% CI 42.2–51.8) of the bacterial isolates investigated. Marco-Fuertes et al. [34] reported that more than 70% of infection-causing *Staphylococcus* spp. strains in dogs exhibited MDR.

As sampling sites are concerned, it was interesting to note that the highest percentage of MDR in our study was detected among isolates from suppurating wounds and abscesses. More than half of the wound isolates showed resistance to eight and nine groups of AMDs. In the study of Lenart-Boron et al. [32], 13.2% of *Enterobacteriaceae* isolated from wounds of small animals and 11.8% of staphylococci were resistant to more than six AMD groups.

Martins et al. [2] provided information on the occurrence of bacteria and yeasts associated with otitis in dogs and cats with alarming rates of multidrug-resistant bacteria: in 57% (99/174) of the bacterial isolates. Among MDR *Pseudomonas spp*, this rate was92% (23/25), *Enterobacteriaceae*: 86.6% (26/30), CoNS: 55% (12/22), *Streptococcus* spp.: 50% (2/4) and CoPS: 41% (32/78). In line with the findings of the present study, 71% of *Streptococcus* spp. isolates from dogs living in the Chattogram Metropolitan Area, Bangladesh were MDR; the majority of them were resistant to five and six classes of AMDs [36].

The emergence of multi-resistant staphylococci, in particular, methicillin-resistant strains, is a critical medical issue in both human and veterinary medicine [5]. In our study, MDR *Staphylococcus* spp. isolates made up 59.3% of all 405 MDR strains. Their share significantly increased in 2023, both in relation to the beginning of the survey period (*p* = 0.002) and to the preceding year 2022 (*p* = 0.008). Skin isolates accounted for 41.7% of all MDR staphylococci (100 out of 240), followed by those from otitis (28.3%, 68 out of 240). Out of 7805 canine clinical samples submitted to a diagnostic laboratory in Tennessee, USA [37] most *Staphylococcus* spp. isolates were from the skin (56.2%), followed by the ears (16.8%) and urine/the urinary bladder (13.3%). This USA study provides evidence about substantial levels of MDR among *S. pseudintermedius* (45.5%) and *S. aureus* (40.9%) isolates from canine samples and increasing temporal trends in MRSP and MRSA occurrence. The authors concluded that temporal changes in antimicrobial resistance patterns among canine staphylococcal isolates make the continued surveillance essential to identifying patterns, trends and newly emerging resistance.

Finally, some limitations of the present retrospective study should be commented upon. The most important limitation is the identification of isolates at the genus level. Although sufficient for the clinical practice and AMR tests, it does not allow a more detailed discussion of the observed trends. Other limitations of the survey include its retrospective character, as well as the lack of complete data on signalment and clinical status of all patients, in particular, history of previous treatment with antimicrobial drugs, bearing in mind that submissions to a microbiological diagnostic laboratory are often associated with failure of therapy.

In conclusion, bacterial resistance to the most conventional antibiotics licensed for veterinary use poses a serious risk for animals and men. The observed ascending and descending AMR against the tested AMDs may be attributed to their more-or-less-frequent usage and resulting different selection pressure. The increasing occurrence of MDR clinical isolates from dogs confirms the importance of regular local monitoring of antimicrobial susceptibility patterns. Despite the listed limitations, this study provides up-to-date epidemiological information that may be useful as guide of future genetic studies on the topic.

## Figures and Tables

**Table 1:** Number of canine isolates from the different microbial pathogens’ groups over the 5-year period of this study.

Groups of Microorganisms	Year of Study					Total
	2019	2020	2021	2022	2023	
*Staphylococcus* spp.	80	92	131	173	214	690
*Streptococcus* spp.	22	18	22	27	24	113
*Enterobacteriaceae*	14	28	23	22	30	117
*Pseudomonas* spp.	13	15	13	10	18	69
Other bacteria *	7	10	15	1	24	57
Yeasts	25	34	34	39	69	201
Total	161	197	238	272	379	1247

* Corynebacterium spp., Pasteurella spp., Enterococcus spp.

**Table 2:** Number of canine isolates from the different microbial pathogens’ groups over the 5-year period of this study in relation to sample origin.

	Throat	Eyes	Nose	Ears	Skin	Wounds	Total
*Staphylococcus* spp.	2	56	8	247	294	83	690
*Streptococcus* spp.	3	21	3	39	25	22	113
*Enterobacteriaceae*	2	6	6	63	21	19	117
*Pseudomonas* spp.	2	10	1	43	8	5	69
Other bacteria *	4	13	8	20	6	6	57
Yeasts	−	−	−	189	12	–	201
Total	13	106	26	601	366	135	1247

* Corynebacterium spp., Pasteurella spp., Enterococcus spp.

**Table 3:** Resistance of canine clinical isolates to commonly tested antimicrobials for the period 2019–2023.

	*Staphylococcus* spp.		*Streptococcus* spp.		*Enterobacteriaceae*		*Pseudomonas* spp.	
Antibiotic	Isolates Tested	% Resistant (95% CI)	Isolates Tested	% Resistant (95% CI)	Isolates Tested	% Resistant (95% CI)	Isolates Tested	% Resistant (95% CI)
Amoxicillin/CA	649	14.9 (12.2–18.2)	104	13.5 (8.2–21.3)	109	19.3 (13–27.7)	53	94.3 (84.6–98.1)
Cephalexin	393	14.8 (11.2–19.1)	49	28.6 (17.9–42.4)	41	51.2 (36.5–65.8)	11	81.8 (52.3–94.9)
Cefovecin	32	34.4 (17.2–61.5)	9	44.4 (18.9–73.3)	7	57.1 (25–84.2)	3	100 (43.9–100)
Cefquinome	416	9.6 (6.9–13.1)	81	19.2 (12.8–27.8)	95	15.8 (9.8–24.4)	53	49.1 (36.1–62.1)
Enrofloxacin	543	28.7 (24.4–33.6)	88	81.8 (72.5–88.5)	100	24 (16.7–33.2)	62	32.8 (22.3–45.3)
Marbofloxacin	426	37.3 (31.8–43.6)	78	85.9 (76.5–91.9)	103	18.4 (12.1–27)	61	31.1 (20.9–43.6)
Levofloxacin	30	23.3 (9.4–48.1)	6	66.7 (30–90.3)	6	16.7 (3.0–56.3)	7	0 (0–35.4)
Orbifloxacin	228	45.6 (37.3–55.3)	36	94.4 (81.9–98.5)	55	36.4 (24.9–49.6)	40	70 (54.6–81.9)
Gentamicin	673	17.1 (14.1–20.5)	111	18.9 (12.7–27.2)	116	13.8 (8.7–21.2)	69	5.8 (2.3–13.9)
Amikacin	303	19.8 (15.1–25.5)	75	64 (52.7–73.9)	83	21.7 (14.2–31.7)	61	14.8 (7.9–25.7)
Neomycin	18	33.3 (12.2–72.6)	5	60 (23.1–88.2)	8	37.5 (13.7–69.4)	6	16.7 (3.0–56.3)
Tobramycin	124	11.3 (6.2–18.9)	31	22.6 (11.4–39.8)	22	4.6 (0.8–21.8)	33	3.0 (0.5–15.3)
Clarithromycin	104	52.9 (39.8–68.8)	13	69.2 (42.4–87.3)	–	–	–	–
Azithromycin	89	52.8 (38.8–70.2)	8	50 (21.5–78.5)	−	−	–	–
Colistin	150	62.7 (50.6–76.7)	21	76.2 (54.9–89.4)	39	74.4 (58.9–85.4)	18	22.2 (9.0–45.1)
Doxycycline	279	36.9 (30.1–44.8)	39	61.5 (45.9–75.1)	25	76 (56.6–88.5)	9	100 (70.1–100)
Lincomycin	123	62.6 (49.4–78.2)	9	44.4 (18.9–73.3)	–	–	–	–
Lincospectin	406	56.9 (49.8–64.7)	82	51.2 (40.6–61.7)	85	84.7 (75.6–90.8)	29	93.1 (78–98.1)
Clindamycin	141	59.6 (47.5–73.8)	20	70 (48.1–85.5)	8	100 (59.8–100)	–	–
Chlorampehicol	294	36.7 (30.1–44.3)	60	25 (15.8–37.2)	46	34.8 (22.7–49.2)	26	73.1 (53.9–86.3)
Rifampin	12	16.7 (2.0–60.2)	–	–	–	–	–	–
ST	243	40.3 (32.7–49.2)	28	46.4 (29.5–64.2)	26	42.3 (25.5–61.1)	5	100 (56.6–100)

CA: clavulanic acid; ST: sulfamethoxazole/trimethoprim. Note: not all isolates were tested against all listed antimicrobials.

**Table 4:** Year-wise resistance rates of *Staphylococcus* spp. clinical isolates to commonly tested antimicrobials for the period 2019–2023.

Antibiotic	Isolates Tested (% Resistant)				
	2019	2020	2021	2022	2023
Amoxicillin/CA	31 (14.9)	27 (17.2)	27 (13.0)	65 (8.9)	94 (20.4)
Cephalexin	36 (8.3)	43 (14.0)	77 (11.7)	125 (11.2)	112 (23.2)
Cefovecin	7 (28.6)	13 (30.8)	10 (50.0)	2 (0)	−
Cefquinome	52 (5.8)	69 (20.3)	101 (6.9)	98 (8.2)	96 (8.3)
Enrofloxacin	66 (31.8)	82 (32.9)	120 (29.2)	129 (24.0)	146 (28.8)
Marbofloxacin	59 (33.9)	67 (40.3)	85 (36.5)	64 (40.6)	151 (36.6)
Orbifloxacin	28 (50.0)	42 (40.5)	29 (48.3)	47 (31.9)	82 (53.7)
Gentamicin	77 (18.2)	88 (26.1)	124 (16.1)	172 (20.3)	212 (10.8)
Amikacin	35 (37.1)	49 (26.5)	65 (12.3)	49 (18.4)	105 (16.2)
Tobramycin	6 (33.3)	20 (5.0)	20 (0)	23 (26.1)	55 (9.1)
Clarithromycin	8 (75.0)	14 (35.7)	22 (63.6)	29 (31.0)	31 (67.7)
Azithromycin	−	9 (55.6)	10 (80.0)	27 (40.7)	43 (53.5)
Colistin	14 (42.9)	28 (53.6)	13 (38.5)	35 (45.7)	60 (86.7)
Doxycycline	34 (55.9)	25 (40.0)	52 (38.5)	87 (28.7)	81 (35.8)
Lincomycin	21 (66.7)	12 (58.3)	14 (71.4)	31 (61.3)	45 (60.0)
Lincospectin	41 (63.4)	55 (60.0)	92 (56.5)	113 (46.9)	105 (63.8)
Clindamycin	15 (73.3)	15 (60.0)	43 (72.9)	59 (47.5)	9 (55.6)
Chlorampehicol	29 (31.0)	35 (34.3)	49 (42.9)	97 (39.2)	84 (33.3)
ST	30 (40.0)	27 (37.0)	27 (63.3)	65 (20.0)	94 (48.9)

CA: clavulanic acid; ST: sulfamethoxazole/trimethoprim.

**Table 5:** Year-wise resistance rates of *Enterobacteriaceae* clinical isolates to commonly tested antimicrobials for the period 2019–2023.

Antibiotic	Isolates Tested (% Resistant)				
	2019	2020	2021	2022	2023
Amoxicillin/CA	11 (45.5)	27 (7.4)	23 (30.4)	20 (15.0)	28 (14.3)
Cephalexin	4 (50.0)	9 (55.6)	11 (72.7)	8 (50.0)	9 (22.2)
Cefquinome	11 (36.4)	25 (20.0)	20 (20.0)	18 (5.6)	21 (4.8)
Enrofloxacin	13 (38.5)	27 (22.2)	22 (40.9)	14 (14.3)	24 (8.3)
Marbofloxacin	14 (35.7)	25 (4.0)	20 (40.0)	16 (18.8)	28 (7.1)
Orbifloxacin	7 (42.9)	14 (21.4)	6 (33.3)	11 (36.4)	17 (49.1)
Gentamicin	14 (14.3)	28 (17.9)	22 (18.2)	22 (9.1)	30 (10.0)
Amikacin	13 (46.2)	19 (31.6)	15 (13.3)	13 (15.4)	23 (8.7)
Colistin	2 (50.0)	10 (90.0)	6 (66.7)	9 (55.6)	12 (83.3)
Doxycycline	3 (66.7)	6 (50.0)	4 (100.0)	5 (80.0)	7 (85.7)
Lincospectin	10 (100.0)	14 (92.9)	20 (65.0)	16 (87.5)	25 (88.8)
Chlorampehicol	1 (0.0)	11 (27.3)	11 (54.5)	14 (42.9)	9 (11.1)
ST	1 (0.0)	4 (75.0)	7 (85.7)	5 (0.0)	9 (22.2)

CA: clavulanic acid; ST: sulfamethoxazole/trimethoprim.

## Data Availability

The data presented in this study are available upon request from the corresponding author.

## Supplementary Materials

The following supporting information can be downloaded at https://www.mdpi.com/article/10.3390/microorganisms12081670/s1, Table S1. Year-wise resistance rates of *Streptococcus* spp. clinical isolates to commonly tested antimicrobials for the period 2019–2023; Table S2. Year-wise resistance rates of *Pseudomonas* spp. clinical isolates to commonly tested antimicrobials for the period 2019–2023; Table S3. Resistance patterns in strains resistant to 7 (n = 14), 8 (n = 5) and 9 (n = 8) groups of antimicrobial drugs (red cells—resistant; green cells—sensitive; white cells—not tested).

## References

[1] Gee N.R., Rodriguez K.E., Fine A.H., Trammell J.P. (2021). Dogs Supporting Human Health and Well-Being: A Biopsychosocial Approach. Front. Vet. Sci..

[2] Martins E., Maboni G., Battisti R., da Costa L., Selva H.L., Levitzki E.D., Gressler L.T. (2022). High rates of multidrug resistance in bacteria associated with small animal otitis: A study of cumulative microbiological culture and antimicrobial susceptibility. Microb. Pathog..

[3] Santaniello A., Sansone M., Fioretti A., Menna L.F. (2020). Systematic review and meta-analysis of the occurrence of ESKAPE bacteria group in dogs, and the related zoonotic risk in animal-assisted therapy, and in animal-assisted activity in the health context. Int. J. Environ. Res. Public Health.

[4] Caneschi A., Bardhi A., Barbarossa A., Zaghini A. (2023). The use of antibiotics and antimicrobial resistance in veterinary medicine, a complex phenomenon: A narrative review. Antibiotics.

[5] Štempelová L., Kubašová I., Bujňáková D., Kačírová J., Farbáková J., Maďar M., Karahutová L., Strompfová V. (2022). Distribution and characterization of staphylococci isolated from healthy canine skin. Top. Companion Anim. Med..

[6] Nocera F.P., Ambrosio M., Fiorito F., Cortese L., De Martino L. (2021). On Gram-positive- and Gram-negative-bacteria-associated canine and feline skin infections: A 4-year retrospective study of the University Veterinary Microbiology Diagnostic Laboratory of Naples, Italy. Animals.

[7] Li Y., Fernández R., Durán I., Molina-López R.A., Darwich L. (2021). Antimicrobial resistance in bacteria isolated from cats and dogs from the Iberian Peninsula. Front. Microbiol..

[8] Marchetti L., Buldain D., Gortari Castillo L., Buchamer A., Chirino-Trejo M., Mestorino N. (2021). Pet and stray dogs as reservoirs of antimicrobial-resistant *Escherichia coli*. Int. J. Microbiol..

[9] Conner J.G., Smith J., Erol E., Locke S., Phillips E., Carter C.N., Odoi A. (2018). Temporal trends and predictors of antimicrobial resistance among Staphylococcus spp. isolated from canine specimens submitted to a diagnostic laboratory. PLoS ONE.

[10] Gómez-Beltrán D.A., Villar D., López-Osorio S., Ferguson D., Monsalve L.K., Chaparro-Gutiérrez J.J. (2020). Prevalence of antimicrobial resistance in bacterial isolates from dogs and cats in a Veterinary Diagnostic Laboratory in Colombia from 2016–2019. Vet. Sci..

[11] Leet-Otley K., Fellman C.L., Wayne A.S., Beaulac K., DeStefano I.M., Chambers K., Marino K.B., Doron S. (2023). Demonstrating the importance of local culture and susceptibility data: Antibiograms from dogs at a veterinary tertiary care center. J. Am. Vet. Med. Assoc..

[12] Petrov V., Mihaylov G., Tsachev I., Zhelev G., Marutsov P. (2013). Otitis externa in dogs: Microbiology and antimicrobial susceptibility. Revue Méd. Vét..

[13] Terziev G., Urumova V. (2018). Retrospective study on the etiology and clinical signs of canine otitis. Comp. Clin. Pathol..

[14] Petrov V., Zhelev G., Marutsov P., Koev K., Georgieva S., Toneva I., Urumova V. (2019). Microbiological and antibacterial resistance profile in canine otitis externa—A comparative analysis. Bulg. J. Vet. Med..

[15] Markey B., Leonard F., Archambault M., Cullinane A., Maguire D. (2013). Clinical Veterinary Microbiology.

[16] CLSI (2018). Performance Standards for Antimicrobial Disk and Dilution Susceptibility Tests for Bacteria Isolated From Animals.

[17] Magiorakos A.P., Srinivasan A., Carey R.B., Carmeli Y., Falagas M.E., Giske C.G., Harbarth S., Hindler J.F., Kahlmeter G., Olsson-Liljequist B. (2012). Multidrug-resistant, extensively drug-resistant and pandrug-resistant bacteria: An international expert proposal for interim standard definitions for acquired resistance. Clin. Microbiol. Infect..

[18] Guardabassi L., Damborg P., Stamm I., Kopp P.A., Broens E.M., Toutain P.L., ESCMID Study Group for Veterinary Microbiology (2017). Diagnostic microbiology in veterinary dermatology: Present and future. Vet. Dermatol..

[19] Rosales R.S., Ramírez A.S., Moya-Gil E., de la Fuente S.N., Suárez-Pérez A., Poveda J.B. (2024). Microbiological survey and evaluation of antimicrobial susceptibility patterns of microorganisms obtained from suspect cases of canine otitis externa in Gran Canaria, Spain. Animals.

[20] Bajwa J. (2023). *Malassezia* species and its significance in canine skin disease. Can. Vet. J..

[21] Tesin N., Stojanovic D., Stancic I., Kladar N., Ružić Z., Spasojevic J., Tomanic D., Kovacevic Z. (2023). Prevalence of the microbiological causes of canine otitis externa and the antibiotic susceptibility of the isolated bacterial strains. Pol. J. Vet. Sci..

[22] Qekwana D.N., Oguttu J.W., Sithole F., Odoi A. (2017). Burden and predictors of *S. aureus* and *S. pseudintermedius* infections among dogs presented at an academic veterinary hospital in South Africa (2007–2012). Peer J..

[23] Awosile B.B., Mcclure J.T., Saab M.E., Heider L.C. (2018). Antimicrobial resistance in bacteria isolated from cats and dogs from the Atlantic Provinces, Canada from 1994–2013. Can. Vet. J..

[24] Yılmaz K.N., Baş B. (2024). Superficial pyoderma in cats and dogs: A retrospective clinical study. Ankara Univ. Vet. Fak. Derg..

[25] Bourély C., Cazeau G., Jarrige N., Leblond A., Madec J.Y., Haenni M., Gay E. (2019). Antimicrobial resistance patterns of bacteria isolated from dogs with otitis. Epidemiol. Infect..

[26] Secker B., Shaw S., Atterbury R.J. (2023). *Pseudomonas* spp. in canine otitis externa. Microorganisms.

[27] Arain M.B., Ambreen L., Muhammad K.F., Muhammad F.H., Ahmed L.S., Salam K.A., Ismail M.M., Zainab L., Ur R.I., Shahzeb A. (2024). Prevalence and characterization of in vitro susceptibility profile of bacteria harvested from otitis externa in dogs. Pak-Euro J. Med. Life Sci..

[28] Kožár M., Hamilton H., Koščová J. (2018). Types of wounds and the prevalence of bacterial contamination of wounds in the clinical practice of small animals. Folia Vet..

[29] Corsini C.M.M., Silva V.O., Carvalho O.V., Sepúlveda R.V., Valente F.L., Reis E.C.C., Moreira M.A.S., Silva Júnior A., Borges A.P.B. (2020). Emergence of multidrug-resistant bacteria isolated from surgical site infection in dogs and cats. Arq. Bras. Med. Vet. Zootec..

[30] Kizerwetter-Świda M., Chrobak-Chmiel D., Stefańska I., Kwiecień E., Rzewuska M. (2024). In vitro activity of selected antimicrobials against methicillin-resistant *Staphylococcus pseudintermedius* of canine origin in Poland. Vet. Med. Sci..

[31] Ludwig C., de Jong A., Moyaert H., El Garch F., Janes R., Klein U., Morrissey I., Thiry J., Youala M. (2016). Antimicrobial susceptibility monitoring of dermatological bacterial pathogens isolated from diseased dogs and cats across Europe (ComPath results). J. Appl. Microbiol..

[32] Lenart-Boroń A., Stankiewicz K., Czernecka N., Ratajewicz A., Bulanda K., Heliasz M., Sosińska D., Dworak K., Ciesielska D., Siemińska I. (2024). Wounds of companion animals as a habitat of antibiotic-resistant bacteria that are potentially harmful to humans-phenotypic, proteomic and molecular detection. Int. J. Mol. Sci..

[33] Moerer M., Lübke-Becker A., Bethe A., Merle R., Bäumer W. (2023). Occurrence of antimicrobial resistance in canine and feline bacterial pathogens in Germany under the Impact of the TÄHAV amendment in 2018. Antibiotics.

[34] Marco-Fuertes A., Marin C., Gimeno-Cardona C., Artal-Muñoz V., Vega S., Montoro-Dasi L. (2024). Multidrug-resistant commensal and infection-causing *Staphylococcus* spp. isolated from companion animals in the Valencia region. Vet. Sci..

[35] Andonova M., Urumova V. (2013). Immune surveillance mechanisms of the skin against the stealth infection strategy of *Pseudomonas aeruginosa*—Review. Comp. Immunol. Microbiol. Infect. Dis..

[36] Deb P., Das T., Nath C., Ahad A., Chakraborty P. (2020). Isolation of multidrug-resistant *Escherichia coli*, *Staphylococcus* spp., and *Streptococcus* spp. from dogs in Chattogram Metropolitan Area, Bangladesh. J. Adv. Vet. Anim. Res..

[37] Lord J., Millis N., Jones R.D., Johnson B., Kania S.A., Odoi A. (2022). Patterns of antimicrobial, multidrug and methicillin resistance among *Staphylococcus* spp. isolated from canine specimens submitted to a diagnostic laboratory in Tennessee, USA: A descriptive study. BMC Vet. Res..

