# Supplementary material for: A Retrospective Study (2019–2023) on the Prevalence and Antimicrobial Resistance of Isolates from Canine Clinical Samples Submitted to the University Veterinary Hospital in Stara Zagora, Bulgaria
Source: Microorganisms. 2024 Aug 14;12(8):1670. doi: 10.3390/microorganisms12081670 (PMC11356874; doi:10.3390/microorganisms12081670)
Supplement: Supplementary file 1 [file microorganisms-12-01670-s001.zip › microorganisms-3139136-supplementary.pdf]

Table S1. Year-wise resistance rates of *Streptococcus* spp. clinical isolates to commonly tested antimicrobials for the period 2019-2023

Antibiotic	Isolates tested (% resistant)				
	2019	2020	2021	2022	2023
Amoxicillin/CA	19 (15.8)	17 (23.5)	20 (20.0)	27 (7.4)	21 (4.8)
Cephalexin	5 (0)	10 (40.0)	12 (33.3)	15 (20.0)	7 (42.9)
Cefquinome	17 (29.4)	14 (35.7)	19 (15.8)	20 (25.0)	11 (18.2)
Enrofloxacin	19 (89.5)	15 (66.7)	20 (85.0)	16 (93.7)	18 (72.2)
Marbofloxacin	17 (94.1)	16 (68.7)	13 (100.0)	13 (92.3)	19 (78.9)
Orbifloxacin	9 (100.0)	5 (80.0)	4 (100.0)	10 (90.0)	8 (100.0)
Gentamicin	21 (9.5)	18 (16.7)	21 (19.0)	27 (25.9)	24 (20.8)
Amikacin	16 (75.0)	11 (72.7)	15 (40.0)	13 (69.2)	20 (65.0)
Tobramycin	4 (50.0)	3 (0)	6 (33.3)	5 (20.0)	13 (15.4)
Colistin	2 (50.0)	4 (50.0)	1 (100.0)	8 (87.5)	6 (83.3)
Doxycycline	3 (33.3)	8 (75.0)	9 (77.8)	15 (53.3)	4 (50.0)
Clindamycin	4 (100.0)	4 (75.0)	7 (71.4)	5 (40.0)	-
Chlorampehicol	12 (25.0)	10 (30.0)	10 (10.0)	15 (26.7)	13 (30.8)
ST	2 (100.0)	2 (100.0)	5 (40.0)	13 (15.4)	6 (83.3)

CA: clavulanic acid; ST: sulfamethoxazole/trimethoprim.

Table S2. Year-wise resistance rates of *Pseudomonas* spp. clinical isolates to commonly tested antimicrobials for the period 2019-2023

Antibiotic	Isolates tested (% resistant)				
	2019	2020	2021	2022	2023
Amoxicillin/CA	10 (100.0)	10 (100.0)	8 (88.9)	9 (100.0)	10 (83.3)
Cephalexin	3 (100.0)	3 (100.0)	2 (50.0)	1 (100.0)	2 (50.0)
Cefquinome	10 (50.0)	12 (91.7)	10 (40.0)	8 (50.0)	13 (15.4)
Enrofloxacin	12 (41.7)	15 (53.3)	13 (15.4)	5 (20.0)	17 (23.4)
Marbofloxacin	12 (33.3)	13 (38.5)	10 (10.0)	8 (50.0)	18 (27.8)
Orbifloxacin	7 (57.1)	11 (72.7)	7 (28.6)	7 (100.0)	8 (87.5)
Gentamicin	13 (7.7)	15 (13.3)	13 (0)	10 (0)	18 (5.6)
Amikacin	11 (27.3)	12 (25.0)	12 (0)	8 (25.0)	18 (5.6)
Tobramycin	6 (0)	7 (14.3)	6 (0)	3 (0)	11 (0)
Colistin	1 (0)	5 (20.0)	1 (0)	6 (33.3)	5 (20.0)
Chlorampehicol	4 (50.0)	5 (80.0)	6 (50.0)	4 (100.0)	7 (87.5)

CA: clavulanic acid.

Table S3. Resistance patterns in strains resistant to seven (n=14), eight (n=5) and nine (n=8) groups of antimicrobial drugs (red cells – resistant; green cells – sensitive; white cells – not tested).

Strains resistant to 7 groups of AMD (n=14)

Sampling site	Isolate	AMCL	CEPH	AMINO	LINC	MACR	TETR	AMPH	FLU	ANSA	POLY	SUL
Ear	*Staphylococcus sp.*	S	R	R	R	R		R	R		S	R
Wound	*Staphylococcus sp.*	R	R	R	R	R	S	R	R			
Wound	*Staphylococcus sp.*	R	R	S	R	R	R	S	R			R
Ear	*Streptococcus sp.*	S	R	R	R	R	S	R	R			R
Wound	*E. coli*	R	R	R	R	R	R		R			
Ear	*Pseudomonas sp.*	R	R	S	R	R	R	R	S			R
Conjunctiva	*Staphylococcus sp.*	R	R	R	R	R	S	R	S			R
Wound	*E. coli*	S	R	S	R	R	R	S	R	R	S	R
Skin	*Staphylococcus sp.*	R	R	R	R	R	S	R	S			R
Skin	*Staphylococcus sp.*	S	R	R	R		S	R	R	R		R
Wound	*Staphylococcus sp.*	R	R	S	S	R	R	R	R			R
Skin	*Staphylococcus sp.*	R	R	S	R	R		R	R			R
Nose	*E. coli*	R	R	R	R	R	R	S	S	S		R
Throat	*E. coli*	R	R	R	R	R	R	S	S			R

Strains resistant to 8 groups of AMD (n=5)

Sampling site	Isolate	AMCL	CEPH	AMINO	LINC	MACR	TETR	AMPH	FLU	ANSA	POLY	SUL
Conjunctiva	*Staphylococcus sp.*	R	S	R	R	R	R	R	S		R	R
Wound	*Proteus sp.*	R	R	R	R	R	S	R	S		R	R
Wound	*Staphylococcus sp.*	R	R	R	R	R	S	R	R		S	R
Wound	*Staphylococcus sp.*	R	R	R	R	R	R	R	S	S	S	R
Skin	*Staphylococcus sp.*	R	R	R	R	R	S	R	R	S		R

Strains resistant to 9 groups of AMD (n=8)

Sampling site	Isolate	AMCL	CEPH	AMINO	LINC	MACR	TETR	AMPH	FLU	ANSA	POLY	SUL
Wound	*Pseudomonas sp.*	R	R	R	R	R	R	R	R			R
Wound	*Staphylococcus sp.*	R	R	R	R	R	R	R	R	S	S	R
Wound	*Staphylococcus sp.*	R	R	R	R	R	R	R	R	S		R
Wound	*Streptococcus sp.*	R	R	S	R	R	R	R	R		R	R
Wound	*E. coli*	R	R	R	R	R	R	R	R		S	R
Wound	*E. coli*	S	R	R	R	R	R	R	R		R	R
Wound	*Streptococcus sp.*	R	R	R	R	R	R	R	S	R		R
Skin	*Staphylococcus sp.*	R	R	R	R	R	R	R	S	S	R	R

Legend: AMCL: beta lactam combination; CEPH: cephalosporins; FLU: fluoroquinolones; AMI: aminoglycosides; MAC: macrolides; POLY: polypeptides; TET: tetracyclines; LIN: lincosamides; AMPH: amphenicols; ANSA: ansamycins; SUL: sulphonamides.
